# A Comparison of Ester‐ and Ether‐Based Bio‐TPUs With Regard to Their Resistance to Environmental Influences in Terms of Their Mechanical and Adhesive Properties

**DOI:** 10.1002/bip.70051

**Published:** 2025-09-20

**Authors:** Marco Klute, Hans‐Peter Heim

**Affiliations:** ^1^ Institute of Material Engineering, Plastic Engineering University of Kassel Kassel Germany

**Keywords:** adhesion, bio‐based TPU, environmental influences, ester‐base, ether‐base, multi‐component injection molding

## Abstract

The demand for bio‐based alternatives to fossil‐based plastics is growing rapidly due to the increasing environmental awareness of consumers and manufacturers, as well as the goal of carbon‐neutral production. There are many promising alternatives that can be obtained from various renewable resources, but their use in series production, especially of technical components, is often hampered by doubts about their usability and, above all, their resistance to environmental influences. The present study is intended to help overcome these obstacles and demonstrate the applicability of bio‐based TPUs in multi‐component technical parts with high bonding requirements. Different polyester and polyether TPUs were used, and their resistance to elevated temperatures and humidity was compared. Both the mechanical properties and the bond strength in bio‐based hard‐soft composites were investigated. It was shown that good to very good bond strengths of approximately 2.5–8 N/mm could be achieved depending on the Shore hardness. The formation of adhesive forces depends on both the type of polyol and its proportion in the TPU. While ether‐based TPU exhibited higher adhesive bond strengths, the strength increases with a higher proportion of soft segments. After storage tests, a decrease in bond strength was observed, mainly due to thermal aging effects and absorption of water molecules, correlating with the change in mechanical properties.

## Graphical abstract

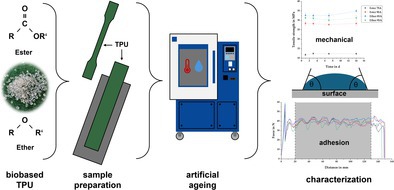

## 1 Introduction

Thermoplastic polyurethanes (TPUs) are versatile materials used extensively in various industries due to their remarkable mechanical properties, flexibility, and resistance to abrasion and chemicals [1, 2, 3, 4]. They are synthesized by the reaction of a polyol with a diisocyanate, whereby the type of polyol used significantly determines the properties of the TPU [5, 6]. These properties can vary between the typical properties of an elastomer and those of a hard thermoplastic. With growing environmental concerns and stringent regulations on fossil‐based products, the demand for sustainable and bio‐based alternatives has surged [7]. Bio‐based TPUs, synthesized from renewable resources, mainly chemically modified plant oils, have emerged as a promising solution. Examples of plants whose oils have so far been used for the synthesis of bio‐polyols are castor oil plant [8, 9, 10, 11, 12, 13], linseed [8, 14, 15], olive [8, 14, 16], rice [8, 14], grapevine [8, 14, 17], sunflower [18, 19], oil palm [20], soybean [19, 20, 21], tung tree [22, 23], passion fruit [15], corn [8], and rapeseed [8, 19]. Compared to other renewable resources that have been exploited to replace crude oil in polymer synthesis, vegetable oils are a promising choice due to their relatively low costs, low toxicity, and inherent biodegradability [8]. When substituting conventional fossil‐based TPUs with the new bio‐based alternatives, it can be seen that the mechanical properties of the end product, such as hardness, tensile strength, and tensile modulus, do not deteriorate [24].

Among bio TPUs, ester‐based and ether‐based types are predominant, each offering distinct properties that make them suitable for different applications. The polyols form the soft segments in the TPUs and have a glass transition temperature that is much lower than the ambient temperature [25]. Depending on which polyol is used, a distinction can be made between ester bonds (CO—O) and ether bonds (C—O). Although the differences between the two modifications are comparably small, the polyester variant shows better mechanical strength properties and higher heat and oil resistance than the polyether variant, making it suitable for applications requiring durability and load‐bearing capacity [26]. The TPUs with polyether polyols are more flexible at lower temperatures and more resistant to hydrolysis, which is advantageous in moist environments or where elasticity is a key requirement [27].

To synthesize bio‐based TPU, the initiator polyol is usually replaced by bio‐based compounds such as sorbitol or sucrose. One example of a bio‐based alternative to fossil‐based polyether polyols is polytrimethylene ether glycol (PO3G). This polyol is produced with different molecular weights in the acid‐catalyzed polycondensation reaction of bio‐based 1,3‐propanediol (PDO) [28]. The use of bio‐based PDO, which is a product of the fermentation of glucose from corn, reduces greenhouse gas emissions from TPU production by approximately 40%–50% compared to fossil‐based PDO [29].

Another example of bio‐based polyols based on PDO is polypropylene succinate glycol (PPS), a product of the polycondensation reaction of PDO and succinic acid. Parcheta et al. were able to demonstrate that this polyester polyol is very well suited for the production of TPU [30, 31]. Other studies have also demonstrated the applicability of other bio‐based polyester polyols such as polybutylene succinate glycol (PBS) [32] and copolyester polyols such as PPS‐co‐PBS [33]. In addition to succinic acid, other bio‐based dicarboxylic acids can also be used for the production of bio‐polyols [32, 34]. The most common of these are sebacic acid, which is obtained from castor oil [35], and azelaic acid, which is obtained by ozonolysis of oleic acids from natural oils and fats [36].

**FIGURE 1:**
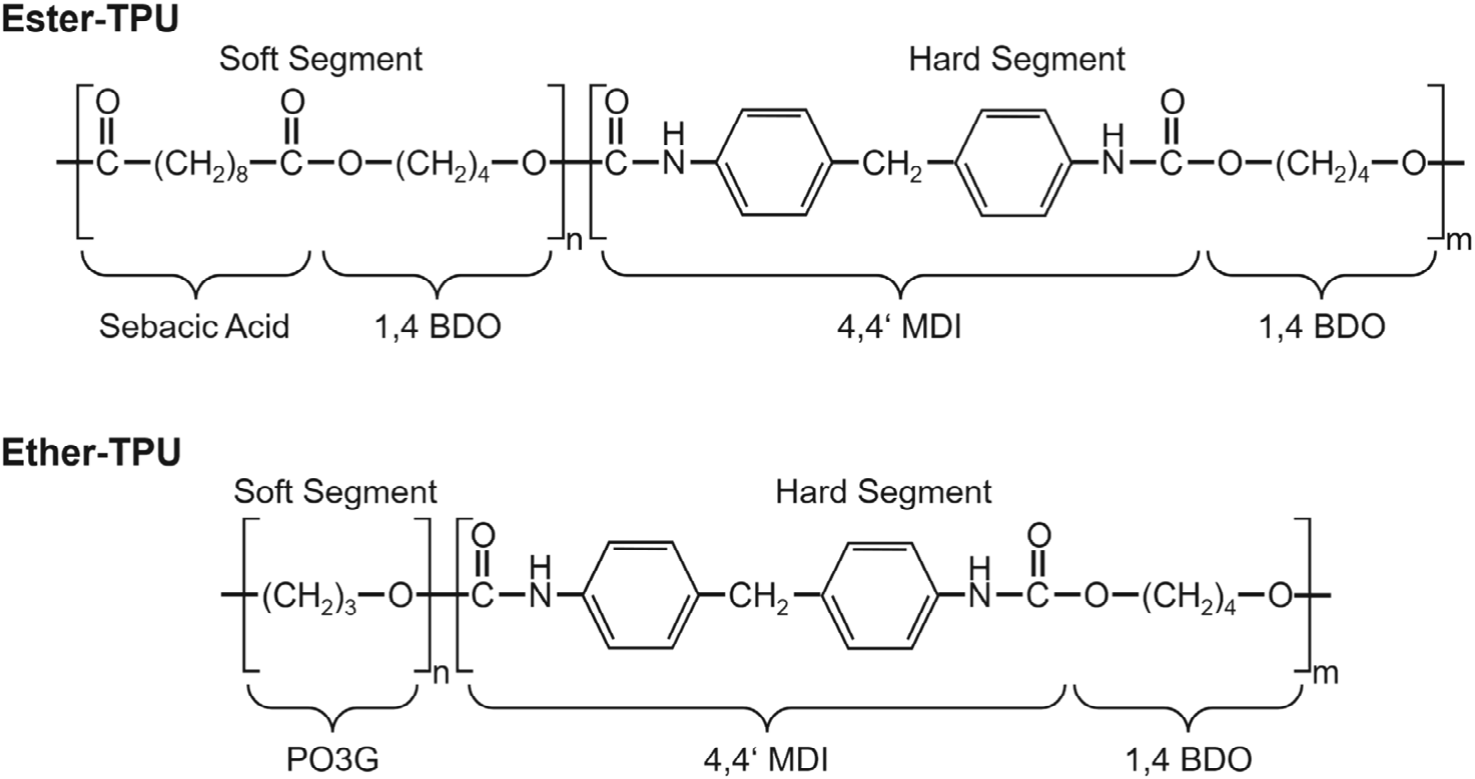
Exemplary chemical structure of biobased ester–and ether–TPU.

Figure 1 shows examples of the chemical structure of ester and ether TPU, whereby the ester TPU shown was synthesized from sebacic acid and the ether TPU from PO3G. In both cases, the hard segment was synthesized from methylenediphenyl isocyanate (MDI) and 1,4‐butanediol (BDO).

Fields of application of TPUs are ranging from footwear [37, 38] and automotive interiors [39, 40, 41] to medical devices [42, 43] and packaging [44, 45], where they are often combined with other plastics, making their adhesive properties a critical factor. Understanding the adhesive performance of bio TPUs is essential for optimizing their use in these applications, ensuring both environmental sustainability and functionality.

One way to combine TPUs with other polymer materials is the advanced manufacturing technique of two‐component (2C) injection molding, also known as multi‐material injection molding. This technique allows the simultaneous or sequential injection of two or more different materials into a single mold, creating form‐fitting components with distinct regions of different materials, each contributing specific properties to the final product. Injection molded plastic–plastic composites are mainly produced to combine material properties, such as color, hardness, or viscosity as well as thermal and electrical properties [46]. With hard‐soft composites in particular, the hard component, which is usually a thermoplastic, absorbs loads, while the soft component, which is usually a thermoplastic elastomer, has a damping or sealing effect or fulfills haptic functions [47, 48]. In order for the components used to fulfill these functions in the material composite, sufficient adhesive bonding is required.

The adhesion mechanisms in 2C injection molding are multifaceted, determined by a complex interplay of material properties, processing conditions, and interfacial phenomena. Understanding these mechanisms is crucial for optimizing the manufacturing process and ensuring the reliability of the produced parts. Adhesion theories can be broadly divided into mechanical, physical, and chemical adhesion phenomena. For polymer‐polymer interfaces, the mechanical interlocking, diffusion, and thermodynamics of the surfaces in particular provide a meaningful basis for describing the resulting adhesive bonds [46]. Each theory offers a different perspective on the factors that contribute to the bonding strength between plastics.

Mechanical interlocking emphasizes the role of surface roughness, interface microstructure, polymer compatibility, viscosity, and processing parameters in determining adhesion [49, 50, 51, 52, 53, 54]. In contrast, diffusion theory attributes adhesion to the interpenetration and entanglement of polymer chains across the interface, a process strongly dependent on molecular weight, temperature, and contact time, particularly in amorphous and semi‐crystalline polymers [55, 56]. From a thermodynamic perspective, Dupré's equation describes the work of adhesion as a function of surface and interfacial tensions [46]; minimal interfacial tension, indicating good adhesion, occurs when polar and disperse fractions of the components are similar [57, 58]. For instance, blending poly(lactic acid) (PLA) with TPU or poly(butylene succinate) (PBS) was shown to reduce interfacial tension to bio‐based ester‐TPUs and enhance adhesion forces by balancing polar and disperse fractions of composite phases [59, 60]. Beyond these intrinsic mechanisms, adhesion is further affected by material and process variables, while surface treatments such as atmospheric plasma [61, 62], UVC irradiation [63, 64], or flame treatment with additive gases [47, 65] are frequently applied to improve interfacial bonding.

In contrast to the studies described above, the focus of this study is not to modify the substrate material or its surface, but to compare the adhesive bonding properties of bio‐based ester‐ and ether‐TPUs. For this purpose, 2C composites are produced from a PLA blend as the hard component and various bio‐based TPUs as the soft component using a 2C injection molding process. PLA was selected as the substrate material because it is the most commonly used bio‐based polymer and, due to its stiffness, is well suited as a hard component for the hard‐soft composites. In order to analyze the resistance of the composites produced to environmental influences, they are stored for different periods of time under different heat and humidity conditions. The changes in both the adhesive and mechanical properties are measured to check the resistance. The results of this study should answer two fundamental research questions, namely how good the adhesive bonding properties of the ester‐ and ether‐based bio‐TPUs are on the PLA substrate and how the TPUs and the material composites react to simulated environmental influences.

## 2 Materials and Methods

### 2.1 Materials

A PLA‐based blend (Bio‐Flex S7514) from FKuR Kunststoffe GmbH (Willich, Germany) was used as the substrate material. This is a blend of PLA with another bio‐based polymer in order to increase the processability in injection molding and the heat deflection temperature of the PLA (due to a valid non‐disclosure agreement, it is not permitted to provide more detailed information on the composition of the blend). Four different TPUs from the same manufacturer were selected as soft components, whereby two ester‐based and two ether‐based types with different Shore hardnesses were used. Table 1 lists the types used, including the abbreviations used, the respective Shore hardness, and the calculated organic content. This bio‐based content of the ester‐based and ether‐based TPU was derived from castor oil and corn, respectively. As these are experimental types from the manufacturer, neither the type designations nor the manufacturer are mentioned. No ester‐based TPU with a hardness of 85 Shore A was available, so a variant with 75 Shore A was used.

**TABLE 1:** Abbreviation and characteristic features of the TPUs used.

Abbreviation	Type	Shore A hardness	Organic content^a^
Ester 75A	Ester‐based	75	49%
Ester 95A	Ester‐based	95	43%
Ether 85A	Ether‐based	85	57%
Ether 95A	Ether‐based	95	49%

^a^ Calculated by the manufacturer.

For the characterization of the surface free energy (SFE), water (H_2_O) and Diiodomethane (CH_2_I_2_) (Sigma‐Aldrich, St. Louis, MO, USA) were used. Their applicability for PLA‐based biopolymers has been shown in numerous studies [59, 60, 66, 67].

### 2.2 Sample Preparation

**FIGURE 2:**
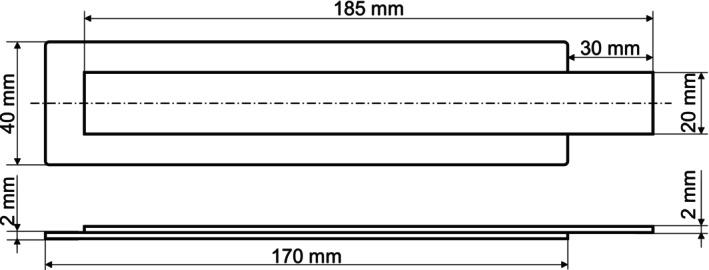
Dimensions of the peel test specimen according to VDI 2019 guideline.

For testing the bonding strength of the composites, 2C peel test specimens were produced according to the test guideline VDI 2019 of the Association of German Engineers (VDI Verein Deutscher Ingenieure e.V., Düsseldorf, Germany), using a 2C injection molding machine (Allrounder 470S from Arburg GmbH + Co KG, Loβburg, Germany) with two injection units. The production process is divided into two successive steps, starting with the injection of the PLA to produce the substrate plate. After a defined cooling time, a movable core opens the second cavity of the mold so that the TPU can be injected directly onto the PLA substrate plate and an adhesive bond is created. The dimensions of the peel specimens are shown in Figure 2, and the parameters used for injection molding are given in Table 2. Prior to injection molding, the TPUs were dried at 80°C, and the substrate material at 60°C for at least 4 h in a dry‐air dryer.

**TABLE 2:** Processing parameters for the injection molding of the 2C peel test specimen.

Process parameter	Injection unit 1 (PLA)	Injection unit 2 (TPU)
Melt temperature in °C	180	220
Mold temperature in °C	30	30
Injection velocity in cm^3^/s	50	50
Injection volume in cm^3^	11	4.5
Packing pressure in bar	600/25	25
Packing time in s	2/1	1
Cooling time in s	15	25

To characterize the mechanical properties of the four different TPUs, tensile test specimens of the type S2 were produced in a four‐cavity mold according to the standard DIN 53504 using an injection molding machine (Allrounder 320C Golden Edition from Arburg GmbH + Co KG, Loβburg, Germany). The processing parameters were set according to Table 3. Only minor adjustments had to be made in order to obtain defect‐free test specimens. To test the change in weight as a function of the storage conditions, tensile test specimens of size A1 (ISO 527) were also produced from three of the four TPUs using the injection molding process (there was not enough material of Ether 95A to produce additional test specimens). A test specimen with a larger volume was selected so that even small changes could be measured. Prior to injection molding, the TPUs were dried at 80°C for at least 4 h in a dry‐air dryer.

**TABLE 3:** Processing parameters for the injection molding of the tensile test specimen.

Process parameter	Ester 75A	Ester 95A	Ether 85A	Ether 95A
Melt temperature in °C	220	220	220	220
Mold temperature in °C	40	50	50	50
Injection velocity in cm^3^/s	50	50	50	50
Switch over point in cm^3^	5.2	5.1	4.8	5.1
Packing pressure in bar	0	1500	1300	1500
Packing time in s	0	1	1	1
Cooling time in s	25	25	25	25

### 2.3 Storage Conditions to Simulate Environmental Influences

In order to test the effects of environmental influences on the bond strength between PLA and TPU, artificial ageing parameters were set. Since the effects of the various environmental influences on the bond strength of the material composite and on the mechanical properties of the respective TPUs are of interest in this study, both the peel and the tensile test specimens were subjected to artificial ageing. All test specimens were stored on the day of manufacture. There were four types of storage supplemented by a reference storage, with four storage periods each. In order to assess the influence of temperature, some of the specimens were stored at 60°C and some at 80°C. In addition, storage at 23°C and 80% relative humidity and at 80°C and 80% relative humidity was carried out in order to be able to draw conclusions about the behavior when exposed to humidity. To be able to assess the behavior of the samples without further influences, there was also a reference storage at 23°C and 50% relative humidity. All these storage types were stored for different lengths of time in the respective environment. Measurements were carried out after 1, 3, 7, and 14 days.

While two UT 6200 convection ovens from the manufacturer Heraeus Holding GmbH (Hanau, Germany) with a test chamber capacity of 180 L were used for increased temperature storage, two different climate chambers (WK1‐340/70, Weiss Technik GmbH, Reiskirchen, Germany and KPK 3526/15, Feutron Klimasimulation GmbH, Langenwetzendorf, Germany) with test chamber capacities of 335 and 400 L were used to store the samples at increased temperatures with varying humidity levels.

The storage conditions are summarized in Table 4, whereby they are given a abbreviations at this point that will be used in the further course. A separate reference batch (Ref(T‐Hu)) had to be produced for T‐Hu because, first, there was not enough material available initially and, secondly, not all test specimens could be produced on the same day. The batches T‐Hu and Ref(T‐Hu) were therefore produced from a different batch of material at a later date, whereby the injection molding parameters had to be adjusted for the production of flawless test specimens compared to the other batches.

**TABLE 4:** Storage conditions and their abbreviations.

Abbreviation	Description	Temperature in °C	Humidity in %
Ref	Standard climate storage (ISO 291)	23	50
Ref(T‐Hu)	Standard climate storage (ISO 291)	23	50
T60	Temperature storage	60	—
T80	Temperature storage	80	—
Hu	Humidity storage	23	80
T‐Hu	Combined temperature and humidity storage	80	80

### 2.4 Peel Tests According to ISO 813‐2 and Guideline VDI 2019

A universal testing machine inspekt Table 5 kN (Hegewald & Peschke Meß‐ und Prüftechnik GmbH, Nossen, Germany) was equipped with a horizontally movable single‐axis test trolley in order to carry out the peel tests in accordance with ISO 813‐2:2024‐02 and the VDI 2019 guideline. The horizontal movement of the test trolley, in which the PLA substrate plate was fixed, ensured a 90° peeling angle of the TPU via a guide pulley. The peel test specimens were stored for at least 16 h at standard conditions according to ISO 291 (23°C, 50% relative humidity) before testing. The test speed was set to 100 mm/min, and five test specimens were tested per material combination, storage condition, and storage time.

**TABLE 5:** Surface tension of the test liquids used for the calculation of the SFE.

Liquid	Surface tension mN/m	Polar component mN/m	Dispersive component mN/m	Source
Water	72.8	51.0	21.8	[68]
Diiodomethane	50.8	2.3	48.5	[69]

To ensure a comparability and to analyze the peel tests performed, the peel resistance *W*_*s*_ is calculated according to Equation (1) as the ratio of the measured force *F* in Newtons and the width *b* in mm of the interface between the two materials of the composites.

(1) $$W_{s}=\frac{F}{b}$$

### 2.5 Microscopy for Analyzing Material Residues After the Peel Test

To visually analyze the material residues remaining on the substrate plates or TPU tabs after the peel tests, microscopic images were taken using a Keyence (Osaka, Japan) VHX‐7000 microscope at 100× magnification.

### 2.6 Tensile Tests According to DIN 53504

The mechanical properties of the TPUs were tested on the above‐mentioned universal testing machine inspekt Table 5 kN, equipped with pneumatic clamps. The type S2 tensile specimens were stored for at least 16 h at standard conditions according to ISO 291 (23°C, 50% relative humidity) before testing. The test speed was 200 mm/min after a preload of 0.1 MPa was reached with a speed of 50 mm/min. The testing machine was additionally equipped with an extensometer, which had a starting distance of 20 mm. Five tests were performed for each TPU, storage condition, and storage time.

### 2.7 Measuremt of the Weight Change

To determine the change in weight due to the different storage conditions, the samples were weighed using an Entris II laboratory balance (Sartorius Lab Instruments GmbH & Co. KG, Göttingen, Germany) of 0.001 g of precision. The weight was first determined immediately before the start of storage in order to have a reference value. On the days of measurement, the samples were removed from the oven or climate chamber, the weight was immediately recorded, and the samples were then returned to the oven or climate chamber. Of the three TPUs considered, 7 samples per storage type (Ref, T60, T80, T‐Hu) were stored over a period of 35 days for Ref, 21 days for T60 and T80, and 14 days for T‐Hu. Since the specimens had different weights at the beginning of storage, the percentage change in weight (∆m) was calculated using Equation (2), where m is the measured weight and $m_{0}$ is the initial weight of each specimen before storage began.

(2) $$∆m\;\left[\%\right]=\frac{m-m_{0}}{m_{0}}∙100$$

### 2.8 Contact Angle Measurement to Calculate the Surface Free Energy

**FIGURE 3:**
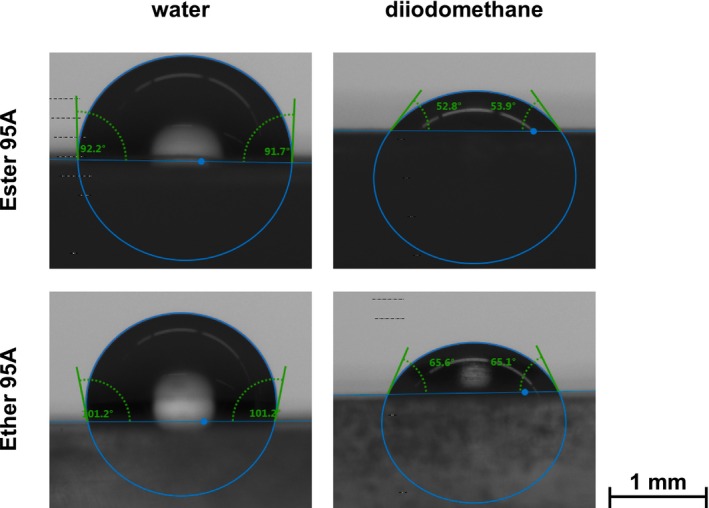
Measured three‐phase angles of water and diiodomethane drops on the surfaces of Ester 95A (Ref) and Ether 95A (Ref) surrounded by air.

In order to thermodynamically describe the prevailing adhesive bond strength in a material composite, the polar and disperse portions of the SFE of the individual materials are important factors, as already mentioned. In order to calculate the SFE, drop shape analyses (DSA) were carried out in this study using the two test liquids, water and diiodomethane, on the contact angle measuring device EasyDrop DSA 20B from Krüss GmbH (Hamburg, Germany). In this method, the test liquids, whose surface tensions are known (Table 5), are applied to the surface of the material to be measured in the form of a drop with a defined volume. The two opposing three‐phase angles between the solid material surface, the liquid drop, and the surrounding gas are determined on the sides of the drop contour, as shown in Figure 3. For water, a contact angle between 1° and 90° represents a hydrophilic surface with good to partial wetting, while 0° corresponds to complete wetting [70, 71]. Surfaces with contact angles of more than 90° are considered as hydrophobic. On every surface, the contact angles of 10 drops of each liquid were measured, and the mean values of those 10 drops were used for the calculation of the SFE. Due to their direct comparability, only the surfaces of Ester 95A and Ether 95A were characterized.

The measured contact angles θ allow the SFE of the solid substrate plate (*σ*_*s*_) to be described using Young's equation (Equation 3). The method of Owens, Wendt, Rabel and Kaelble (OWRK) extends the relationship between the SFE of the solid and the surface tension of the liquid droplet (*σ*_*l*_) by the dispersive (*σ*_*s*_^*D*^ and *σ*_*l*_^*D*^) and polar (*σ*_*s*_^*P*^ and *σ*_*l*_^*P*^) portions of the SFE of the two materials as shown in Equation (4), with $\sigma_{\mathrm{sl}}$ being the interfacial tension between the two materials. OWRK is one of the most common methods for calculating the SFE of polymers [72, 73, 74].

By combining the two equations and using the measured contact angles of the two liquids, the SFE of the plastics used can be calculated, divided into its disperse and polar fractions.

(3) $$\sigma_{s}=\sigma_{\mathrm{sl}}+\sigma_{l}∙\cos\theta$$

(4) $$\sigma_{\mathrm{sl}}=\sigma_{s}+\sigma_{l}-2\left(\sqrt{\sigma_{s}^{D}∙\sigma_{l}^{D}}+\sqrt{\sigma_{s}^{P}∙\sigma_{l}^{P}}\right)$$

### 2.9 Differential Scanning Calorimetry (DSC) to Investigate the Change in Crystallinity

For thermal characterization, a DSC measurement was performed using the Q1000 module from TA Tools (New Castle, USA). The samples were heated at a heating rate of 10 K/min from 0°C to 250°C, then cooled at a cooling rate of 10 K/min to 0°C, and then reheated to 250°C at the same heating rate. Since the thermal history can be analyzed during the first heating, only this is evaluated in this study. The heat flow was recorded over time, as this allows endothermic and exothermic thermal effects, such as the glass transition and the melting of the crystalline areas, to be mapped. The melting enthalpy Δ*H* is defined here as the integral of the corresponding peak. This can be used to calculate the crystallinity of the material, whereby the theoretical enthalpy of the 100% crystalline material must be known as a reference. Since this is not known for the TPU used here, only the recorded melting enthalpy is analyzed.

### 2.10 Fourier Transform Infrared Spectroscopy (FTIR) for Analyzing Intensity Changes of Polar Groups

For the spectroscopic analysis of the atomic bonds contained in the material, FTIR measurements were performed using the IRAffinity‐1S from Shimadzu (Kyōto, Japan), equipped with a germanium crystal. The measurements were performed in a range from 4000 to 600 cm^−1^ using attenuated total reflection (ATR).

## 3 Results

### 3.1 Adhesive Bond Strength of the PLA‐TPU Composites as a Function of Environmental Influences

**FIGURE 4:**
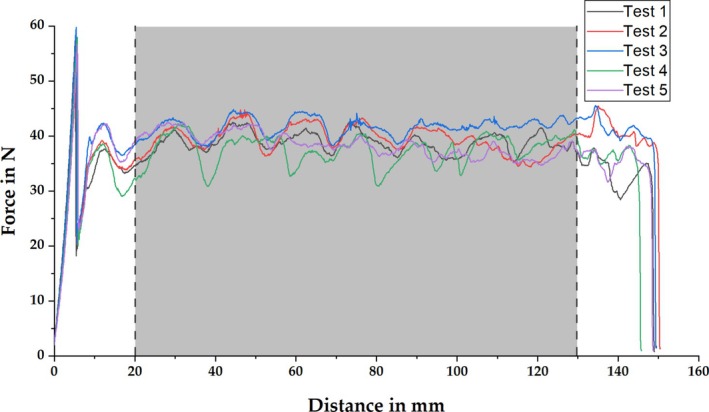
Recorded peel forces of the five test pieces (Test 1–Test 5) of Ester 95A after 1 day of storage at 80°C including limits (dashed lines) for calculating the mean value of the forces.

Before the peel resistance can be calculated from the recorded peel test force curves according to Equation (1), the force curves must be adjusted. Figure 4 shows an example of the force curves for Ester 95A after 1 day of storage at 80°C. It can be seen that there is a comparatively large increase in force before uniform peeling occurs. To ensure that this and similar effects that occur at the end of the specimen do not distort the measurement results, the mean value of the force curve is calculated within defined limits, which are shown in Figure 4 as vertical dashed lines for this batch as an example. Due to the different behavior of the materials and the storage conditions used, these limits were adjusted individually for each batch. The peel strength was calculated from the resulting average peel force values.

**FIGURE 5:**
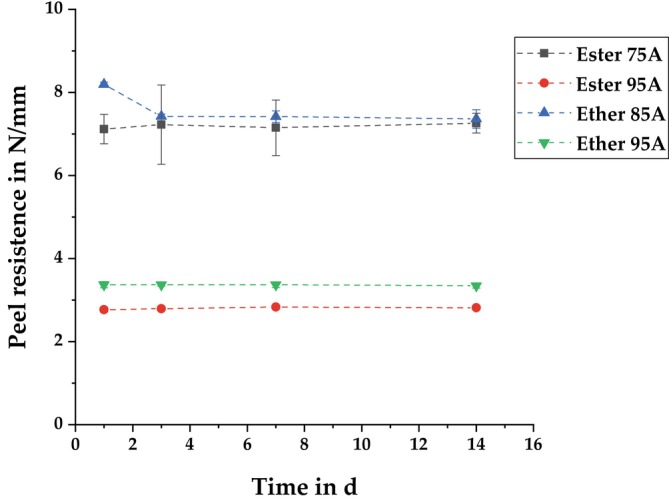
Comparison of the average peel resistance (maximum peel resistance for Ester 75A) of the different TPUs after storage at standard climate conditions.

Figure 5 compares the reference batches of PLA‐TPU composites. These results show a dependence of the peel resistance on the Shore hardness. While the peel resistance of the ether‐based TPU with a Shore A hardness of 95 is approximately 3.4 N/mm on average, the peel resistance of the TPU with 85 Shore A is more than twice as high. The comparison of the two 95 Shore A TPUs shows that the ether‐based TPU has a 21.4% higher peel strength than the ester‐based TPU. In contrast to the other TPUs, the ester‐based TPU with a Shore A hardness of 75 did not peel because the TPU tap failed cohesively before the interface of the composite could fail adhesively. Such cohesive failure always occurs when the adhesive bond strength is higher than the cohesive tensile strength of the TPU. Since there is a correlation between Shore hardness and tensile strength [75], it can be expected that, with the same bond strength, cohesive failure will occur at lower Shore hardnesses, while TPUs with higher Shore hardness can be peeled off adhesively without cohesive failure.

**FIGURE 6:**
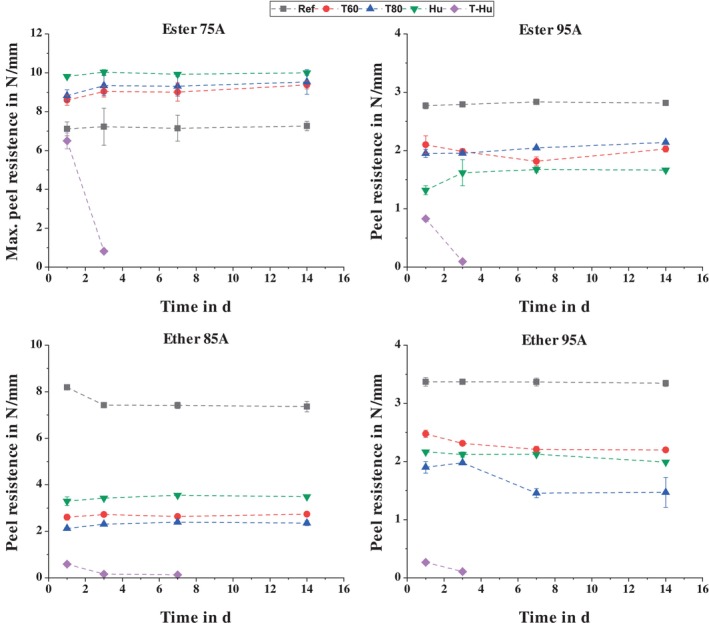
Comparison of the peel resistance of the TPUs as a function of storage condition and time.

The effects of the different storage conditions on the four material combinations considered are shown in Figure 6. With the exception of Ester 75A, where only the maximum forces that caused the TPU tab to fail are shown, all storage conditions resulted in a reduction in bond strength for all TPUs. As expected, combined temperature and moisture storage had the greatest effect on bond strength. In this case, the TPU tab detached from the PLA substrate after 7 days (14 days for Ether 85A) for all material combinations, even for Ester 75A, and peel tests could not be performed. The temperature‐induced increase in water absorption causes the water to diffuse into the interface of the material composites, resulting in the dissolution of the adhesive bond. In addition, there were very pronounced signs of aging on the PLA‐based substrate plates, as some of them disintegrated during storage. Due to these two effects, peel tests could no longer be carried out for these batches.

**FIGURE 7:**
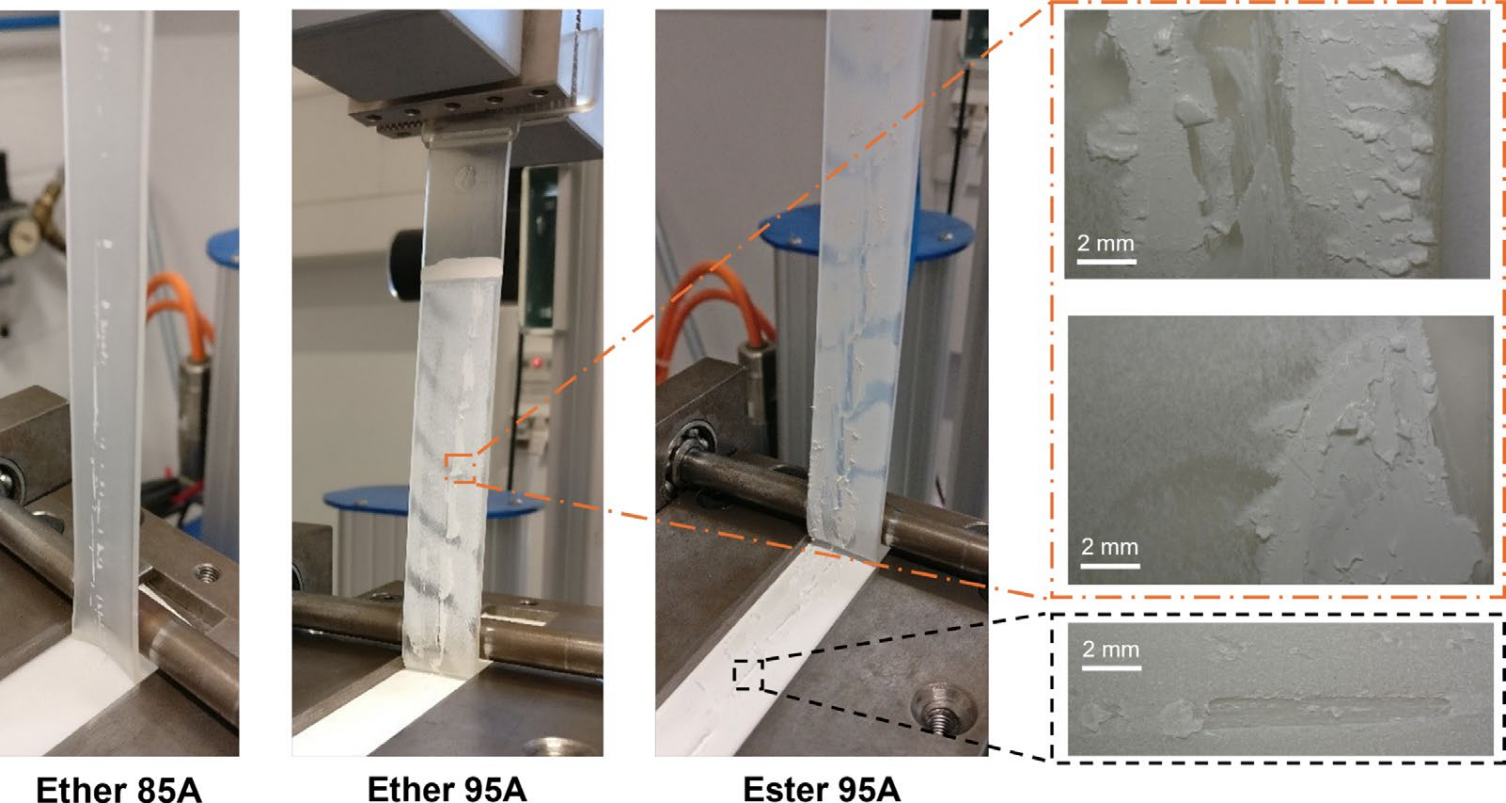
Residue of substrate material observed on TPU tabs during peel (no residue on Ether 85A).

During the peel tests, no residue of the TPU was found on the surface of the substrate plates. However, for the two TPUs with a hardness of 95 Shore A, residues of the substrate material were observed on the peeled TPU tabs. Although Ether 85A showed the highest adhesion during peeling, no such residue was observed with this material. Figure 7 shows an example of the three TPUs mentioned during the peeling process to illustrate the residues. The lower microscope image (Figure 7, right) shows that pieces were torn out of the substrate plate during the peel tests and remain on the TPU tab. The upper two images show laminar residues on the TPU tabs.

### 3.2 Mechanical Properties of the Bio‐Based Ester and Ether TPU as a Function of Environmental Influences

**FIGURE 8:**
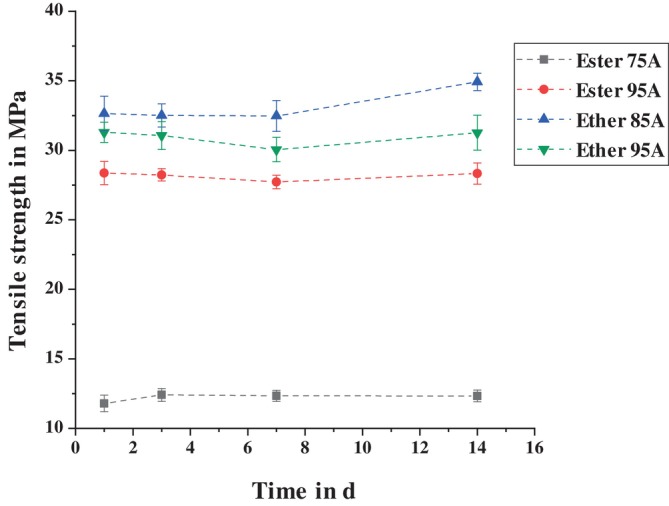
Comparison of the tensile strength of the different TPUs after storage at standard climate conditions.

Analogous to the results of the peel tests, the four different TPUs are first compared with each other. Figure 8 shows the tensile strength of the materials over the storage period in a standard climate. It can be seen that the ester‐based TPU with a Shore A hardness of 75 has the lowest tensile strength. This is due to the fact that this TPU is the only one that contains a plasticizer to reduce the Shore hardness. The comparatively low tensile strength explains the cohesive failure of the Ester 75A TPU tab in the peel tests.

Both ether‐based TPUs have higher tensile strengths compared to the ester‐based TPU with a Shore hardness of 95 Shore A. This does not correspond to the findings of Jin et al. [27] in their study. There, ester‐based TPUs with the same Shore hardness exhibited higher tensile strength than ether‐based TPUs. However, different base materials were used for TPU synthesis in the study, and it was shown that different ratios of hard and soft segments in the TPU are required to achieve the same Shore hardness. For the TPU considered here, the chemical compositions and the ratios between hard and soft segments are not known, which is why it cannot be clearly deduced why the two ether‐based TPUs have higher tensile strength than Ester 95A.

**FIGURE 9:**
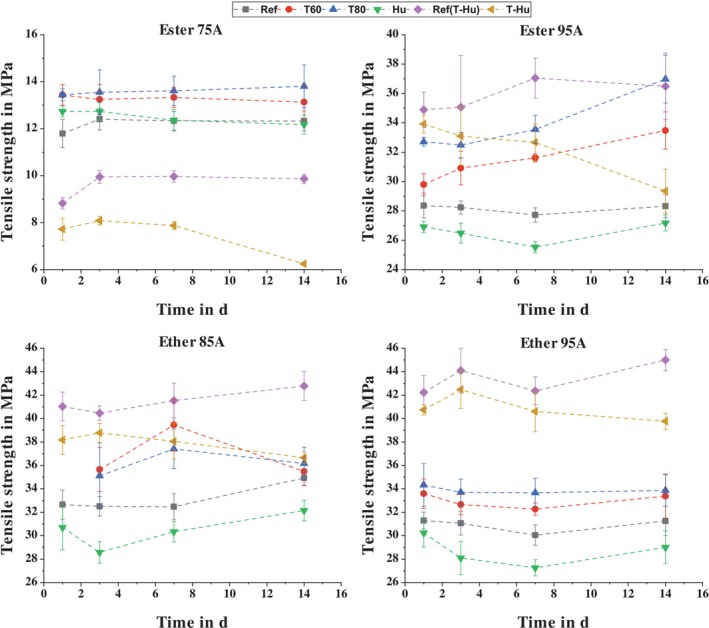
Comparison of the tensile strength of the TPUs as a function of storage condition and time.

In contrast to the ester‐based TPUs, the tensile strength of the ether‐based TPUs appears to decrease with increasing Shore hardness and thus decreasing long‐chain diol content. This contradicts theory and other studies [76, 77]. As mentioned above, not all test specimens could be produced from the same batches of material and in 1 day, so a second reference batch of each material was produced for the storage condition T‐Hu. In this batch, the tensile strengths measured are consistent with theory, as the tensile strength increases with increasing Shore hardness. The second reference batch is shown in Figure 9 together with the results of the different storage conditions. The difference between the two reference batches of the respective TPU can be explained by material batch deviations and different process conditions during injection molding. Due to material and environmental influences (e.g., varying pellet temperature and residual moisture, different air temperature and humidity), the processing parameters had to be adjusted in order to produce flawless test specimens. Frick et al. [78] demonstrated that processing parameters in the injection molding of TPU significantly influence the resulting material properties. In the case of an ester‐based TPU, a reduction in the processing temperature led to a detectable agglomeration of the hard segments, which in turn led to an increase in the melting temperature and stiffness. The observed deviations between the reference batches in this study indicate that there are influencing factors along the processing chain, from material synthesis, storage, drying, and processing, that impair the reproducibility of the results. However, since the results of this study can be interpreted on the basis of the respective reference batches, this does not diminish the significance of the findings.

Apart from small variations, the tensile strength of all the materials considered appears to remain constant over the 14‐day storage period in standard conditions, as expected.

As mentioned above, Figure 9 shows the tensile strengths of the four TPUs in separate graphs as a function of storage type and duration. The Ref batch serves as the reference (standard climate storage) for temperature storage (T60 and T80) and humidity storage (Hu). Ref(T‐Hu) is the reference for combined temperature and humidity storage (T‐Hu).

**FIGURE 10:**
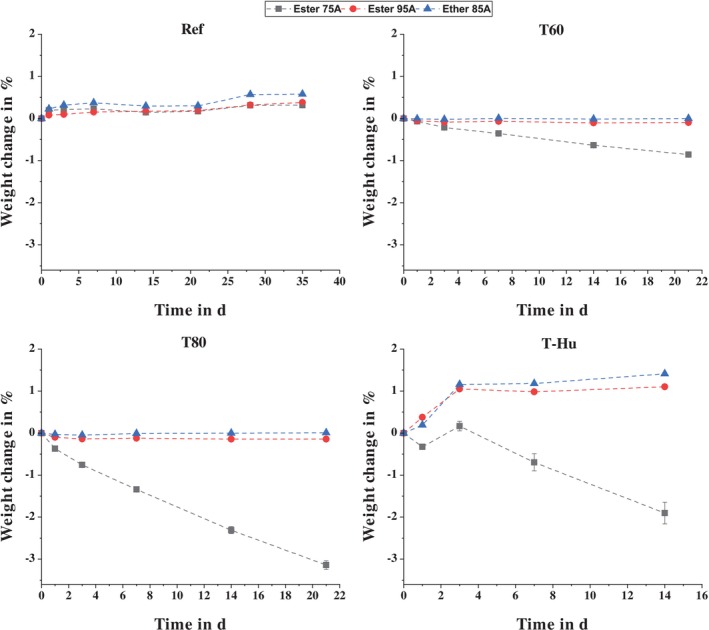
Measured percentage weight change as a function of storage conditions and duration.

Looking first at the effect of temperature storage on the tensile strength of the TPUs, it can be seen that it always results in an increase in tensile strength compared to the reference. With the exception of the ether‐based TPU with a Shore A hardness of 85, the tensile strengths at a storage temperature of 80°C are always higher than those at 60°C. However, the deviation for Ether 85A is burdened with a high standard deviation, so there is no statistical significance here. Based on these results, it can be said that tensile strength increases with increasing temperature. This effect was also observed by Boubakri et al. [79] and was attributed to a loss of volatiles (water molecules, plasticizer), which reduces the plasticity of the material, and oxidation reactions in the polymer causing molecular recombination. Some studies have shown that the mechanical properties of TPU are susceptible to temperature storage and that thermally induced chain scission and cross‐linking occur [80, 81]. However, for short storage periods of less than 6 months, the effect of volatile loss predominates over chain scission and cross‐linking, which is also reflected in a reduction in the weight of the test specimens [79]. Therefore, the weight loss was calculated as described by Equation (2) and is shown in Figure 10. Due to those results, the change in tensile strength can also be correlated to the loss of volatiles as described by Boubakri et al.

With the exception of the ester‐based TPU with a Shore A hardness of 75, the effects of moisture storage at ambient temperature (Hu) are very similar. The tensile strength initially decreases and then increases over time in line with the reference, always remaining at a lower level than the reference. The decrease in tensile strength can be explained by the absorption of water molecules, which act as a plasticizer. This effect was also described by Atiqah et al. [82]. Boubakri et al. [83] also found that the absorption of water molecules depends significantly on the storage temperature. At higher temperatures, the mobility of the chains is increased, which allows the water molecules to diffuse more quickly into the chain interspaces and absorb a greater amount of water. The combination of temperature and humidity therefore has a very pronounced effect on the mechanical properties of TPUs, overshadowing the effects of aging. This can also be seen in the results of this study, as both the percentage decrease in tensile strength and the increase in weight are greatest with combined temperature and humidity storage (T‐Hu). In contrast to humidity storage at ambient temperature (Hu), the tensile strength decreases continuously during combined storage. Compared to the corresponding reference, the tensile strength of Ester 75A, Ester 95A, Ether 85A, and Ether 95A decreased by 36.66%, 19.57%, 14.31%, and 11.63%, respectively, after 14 days of combined storage.

**FIGURE 11:**
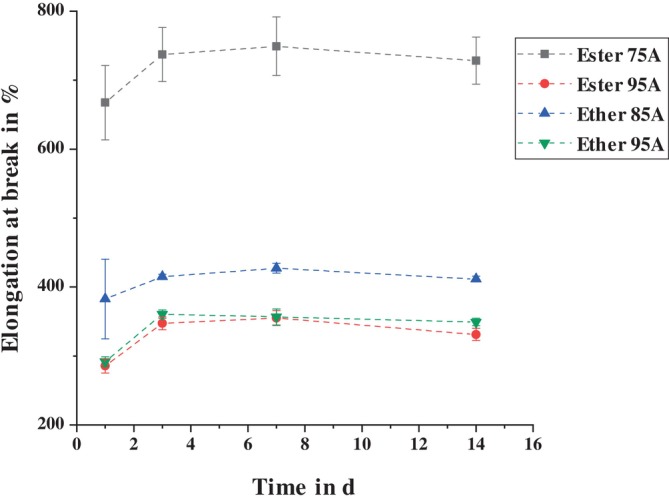
Comparison of the elongation at break of the different TPUs after storage at standard climate conditions.

The behavior described for tensile strength is also partially reflected in the elongation at break of the TPUs. Figure 11 shows the elongation at break of the four different TPUs. The ester‐based TPU with a Shore A hardness of 75 has by far the highest elongation at break. The two TPUs with a Shore A hardness of 95 give similar results although, as expected, their elongation at break is lower than that of the ether‐based TPU with a Shore A hardness of 85. For all four TPUs, the elongation at break initially increases and then decreases over time, although this decrease is not statistically significant over the time period considered.

**FIGURE 12:**
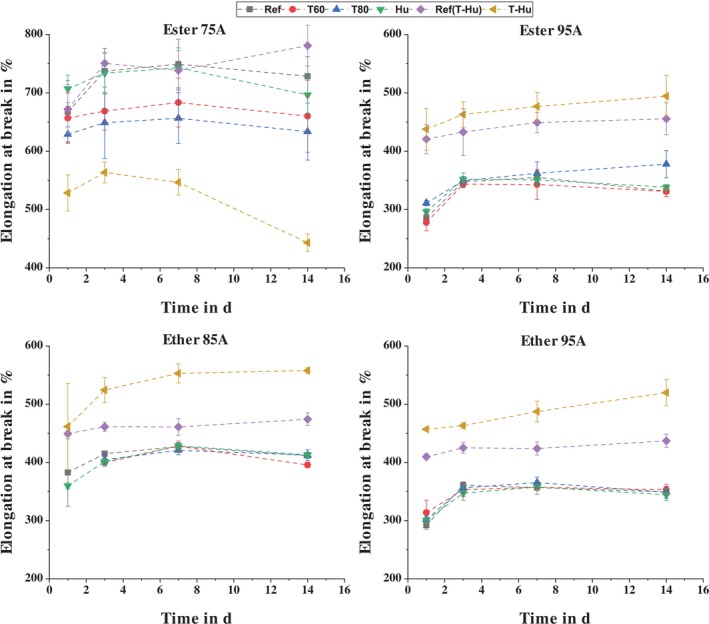
Comparison of the elongation at break of the TPUs as a function of storage condition and time.

The decrease in elongation at break due to temperature storage observed by Boubakri et al. [79] is only observed in the results of this study for the ester‐based TPU with a Shore A hardness of 75 (see Figure 12). The changes in elongation at break of the other TPUs due to the effect of elevated temperatures are very small and not statistically significant. However, it should be noted that Ester 75A, unlike the other TPUs, contains a plasticizer. As a result of storage at elevated temperatures, this plasticizer may have diffused, which would cause a reduction in elongation at break. This diffusion of the plasticizer can be seen very clearly in the significant weight loss (Figure 10).

Although an uptake of water molecules was observed due to the increase in weight of the test specimens, which led to a reduction in tensile strength, the elongation at break was not significantly affected by this. Only the combination of temperature and moisture storage leads to an increase in elongation at break because a greater amount of water molecules has been absorbed. However, this only applies to the three TPUs without plasticizer. In the case of Ester 75A, the combined storage resulted in a significant reduction in elongation at break, which correlates with the change in weight. While the other TPUs increased significantly in weight due to the combined storage, the weight of the Ester 75A samples decreased significantly. It can be assumed that although water is absorbed, the diffusion of the plasticizer (seen during storage at 80°C) has a greater effect on the weight, which is why it decreases overall.

### 3.3 SFE of the TPU as a Function of Environmental Influences

**FIGURE 13:**
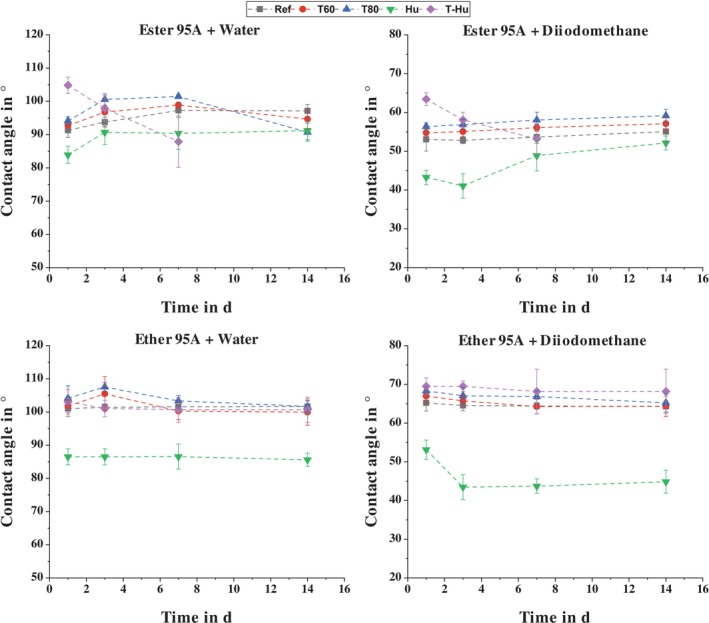
Contact angles of water and diiodomethane drops on Ester 95A and Ether 95A surfaces as a function of storage condition and time.

Figure 13 shows the contact angles of water and diiodomethane on the surfaces of Ester 95A and Ether 95A, averaged from at least 10 drops per liquid, as a function of the type and duration of storage. In the case of Ester 95A, it is noticeable that the contact angle of water increases continuously during the first 7 days for reference storage, storage at elevated temperatures, and humidity storage at ambient temperature, while it decreases significantly for combined storage (since after 7 days of storage no functional 2C test specimens were left, no more tests were carried out for T‐Hu). From the seventh day, a significant decrease in contact angle is observed during temperature storage. This decrease is greater with increasing temperature. For diiodomethane, on the other hand, the angle increases continuously for all types of storage except combined storage. The height of the angle is the same for both liquids depending on the type of storage. Moisture storage at ambient temperature has the lowest angle, while temperature storage increases the angle compared to the reference. The higher temperature also leads to larger angles.

Ether 95A shows similar behavior. Moisture‐only storage gives by far the lowest contact angles, while temperature storage slightly increases the angles compared to reference storage. However, combined storage does not show a significant decrease in angle as a function of storage time. The values are almost constant for all types of storage, with the contact angle of water initially increasing with temperature storage and then decreasing again with time.

Using the averaged contact angles of the two liquids, the SFE of the TPUs was calculated according to the method of OWRK (Equation 4) as a function of the type and duration of storage. The calculated values of the SFE (*σ*_*s*_), divided into the polar (*σ*_*s*_^*P*^) and disperse (*σ*_*s*_^*D*^) components, are shown in Table 6. During the reference storage, it can be observed that the SFE decreases by about 1 mN/m for Ester 95A, while it increases by about 0.6 mN/m for Ether 95A. For the ester‐based TPU, the decrease is due to a reduction in the polar fraction, while the disperse fraction remains the same; for the ether‐based TPU, the increase is due to an increase in the disperse fraction, while the polar fraction decreases slightly.

**TABLE 6:** Calculated SFE (*σ*_*s*_ in mN/m) divided into its polar (*σ*_*s*_^*P*^ in mN/m) and disperse (*σ*_*s*_^*D*^ in mN/m) components as a function of the type and duration of storage.

	Days	Ref			T60			T80			Hu			T‐Hu		
		*σ*_*s*_	*σ*_*s*_^*P*^	*σ*_*s*_^*D*^	*σ*_*s*_	*σ*_*s*_^*P*^	*σ*_*s*_^*D*^	*σ*_*s*_	*σ*_*s*_^*P*^	*σ*_*s*_^*D*^	*σ*_*s*_	*σ*_*s*_^*P*^	*σ*_*s*_^*D*^	*σ*_*s*_	*σ*_*s*_^*P*^	*σ*_*s*_^*D*^
Ester 95A	1	32.57	1.85	30.72	31.59	1.62	29.97	30.67	1.43	29.24	38.15	3.12	35.03	27.12	0.16	26.97
	3	32.72	1.19	31.52	31.51	0.76	30.74	30.77	0.31	30.46	39.2	0.98	38.21	29.77	0.77	29.00
	7	32.44	0.57	31.86	31.07	0.48	30.59	30.13	0.26	29.87	34.9	1.67	33.22	32.74	2.94	29.8
	14	31.55	0.69	30.85	30.26	1.37	28.89	29.35	2.76	26.59	33.09	1.77	31.31			
Ether 95A	1	25.62	0.73	24.89	24.63	0.74	23.89	23.96	0.46	23.5	32.94	3.44	29.5	23.17	0.71	22.46
	3	26.06	0.63	25.43	25.7	0.19	25.51	25.13	0.08	25.05	37.86	2.27	35.6	23.14	1.1	22.04
	7	26.14	0.59	25.54	26.15	0.8	25.35	24.76	0.54	24.21	37.74	2.27	35.47	23.89	1.05	22.84
	14	26.2	0.58	25.63	26.11	0.86	25.24	25.64	0.63	25.01	37.21	2.71	34.5	23.89	1.05	22.84

The effects of storage at elevated temperatures can be seen in the initial decrease in polarity for both TPUs, followed by an abrupt increase during storage. In particular, at 80°C, the polarity value of Ester 95A clearly exceeds the initial value by approximately 93% after 14 days of storage. As mentioned above, in TPU, as in other polymeric materials, the influence of temperature causes the polymer chains to scission and crosslink [80, 81]. As a result, new polar groups such as carbonyls can form at the resulting chain ends, leading to an increase in polarity [84]. In addition, aging at elevated temperatures increases chain mobility, causing rearrangement and reorganization of the polymer chains. This can result in larger spherulites in the hard segment of the TPU, while a more ordered structure is created in the soft segment due to a realignment of the broken chains (due to scission) [85]. Both effects lead to an increase in the degree of crystallinity, which also influences the SFE [46].

While temperature storage leads to a significant change in the polar and disperse components of the SFE, while the latter remains almost unchanged; storage at elevated humidity also has an effect on the level of the total surface energy. The SFE exhibits significantly higher values in humidity storage at ambient temperature compared to the other types of storage. The highest surface polarity values are also obtained with this type of storage. This effect can be explained by the absorption of polar water molecules, as evidenced by the increase in weight.

In the case of combined temperature and humidity storage, the influence of temperature initially appears to be predominant, as both the polar and disperse fractions of SFE in Ester 95A show a comparatively low value after 1 day of storage. During storage, however, the values increase significantly again, which can be explained by the uptake of water molecules and the simultaneous formation of polar groups at the chain ends.

### 3.4 Change in Crystallinity and Polar Edge Groups as a Result of Temperature Storage

**FIGURE 14:**
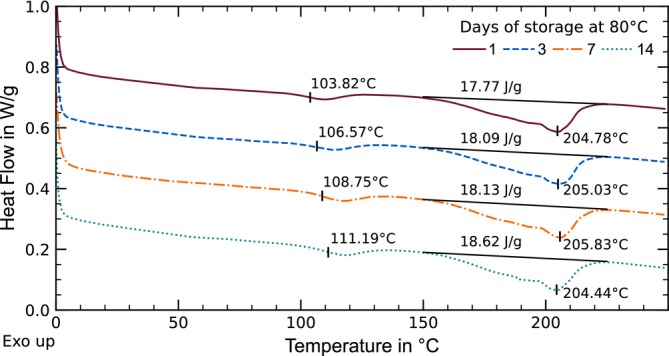
DSC thermogram of Ester 95A stored at 80°C as a function of storage time (the curves have been shifted for better visualization).

As already described, crystallinity influences both the SFE and the adhesive bond strength [46]. In general, it has been proven that higher surface crystallinity leads to higher SFE [86]. To analyze the change in crystallinity of Ester 95A as a function of temperature storage, the first heating curves of the DSC measurements were evaluated. These can be seen in Figure 14, where the glass transition temperature shown at approx. 104°C–111°C corresponds to the hard segments of the TPU and the melting peaks correspond to the soft segments. The degree of crystallinity can be qualitatively determined based on the calculated enthalpy during melting of the crystalline structures. It can be seen that crystallinity increases continuously with increasing storage time. Compared to the value after 1 day of storage, crystallinity has increased by approx. 5% after 14 days of storage. This effect correlates with the results of the mechanical characterization, as tensile strength has increased with increasing storage time.

In addition to the change in melting enthalpy, there is also a shift in the crystallite melting temperature in a positive direction, which indicates an increase in the size of the crystalline structure. The glass transition temperature of the hard segments also shifts, which indicates better bonding of the hard segments to the soft segments [87].

In summary, it can be concluded that the crystallinity of the soft segments increases with temperature storage, which explains the change in mechanical properties. The increase in crystallinity also influences the SFE, but no direct correlation can be identified, as it is influenced by a variety of factors and the effects appear to overlap.

**FIGURE 15:**
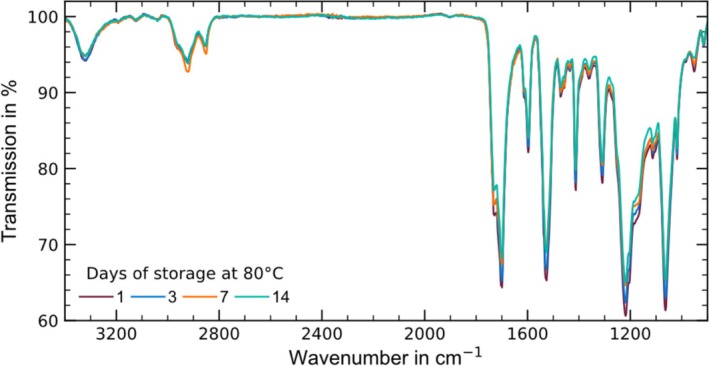
FTIR spectrograms of Ester 95A stored at 80°C as a function of storage time.

SFE is mainly influenced by the polar oxygen‐containing edge groups near the surface. One method for detecting such oxygen‐containing compounds is FTIR‐ATR spectroscopy. Figure 15 shows the spectrograms of Ester 95A samples stored at 80°C in the wavenumber range from 3400 to 900 cm^−1^. Characteristic peaks of TPU can be found at the wavenumbers 3330 cm^−1^ (stretching vibration of NH in the urethane groups [88, 89]), 2935 and 2850 cm^−1^ (asymmetric and symmetric CH_2_ vibration [89]). Only minor changes occur in these wavenumber ranges during temperature storage. Only the measurement after a storage period of 7 days shows an increase in the CH_2_ peaks.

Characteristic wave numbers for oxygen bonds contained in TPU are 1740 cm^−1^ (free C=O bonds [90, 91]), 1700 cm^−1^ (hydrogen bonded C=O bonds [90, 91]), 1220 cm^−1^ (C—O—C stretching vibrations [88]), and 1185–1050 cm^−1^ (C—O stretching vibrations [61]). For all of these wave numbers, there is a decrease in the peaks during temperature storage, which indicates a reduction in oxygen‐containing bonds. The same applies to nitrogen‐containing bonds at the wave numbers 1530 cm^−1^ (N—H bending vibration [88]) and 1310 cm^−1^ (C—N stretching [88]). The decrease in polar groups explains the reduction in SFE and the decrease in adhesive bond strength.

Yuan et al. [90] were able to demonstrate that at temperatures above 80°C, irregular hydrogen bonds (C=O, 1700 cm^−1^) in imperfect crystalline parts dissolve, which increases chain mobility. This allows more regular hydrogen bonds and thus perfect crystalline parts to form, which explains the increase in crystallinity. Overall, the higher chain mobility seems to cause the polar groups to migrate from the edge area to the interior of the sample.

## 4 Discussion

When comparing the different TPUs considered, it should be noted that all four TPUs showed good to very good adhesion to the PLA‐based substrate. Therefore, they can all be used for the production of multi‐component bio‐based hard‐soft composites in multi‐component injection molding. In the case of Ester 75A, the adhesive bond strength was even higher than the tensile strength of the TPU, which is why only cohesive failure of the TPU tab occurred in the peel tests. Therefore, it can be assumed that the bond strength is at a similar, if not higher, level than that of Ether 85A. Accordingly, it is likely that Ester 75A has the highest bond strength of the four material combinations tested.

Since neither the substrate material of the hard‐soft composites nor the processing properties were changed in this study, the composition of the TPUs must cause the difference in bond strength. Therefore, it is important to note that the TPUs differ in two aspects. First, there is a difference in the proportion of soft segments. This must decrease with increasing material hardness so that the materials retain their specified flexibility. Second, there is a difference in their polyol groups, which can influence adhesion due to their different structure. There is a distinction between polyester and polyether polyol groups. In principle, these two aspects correlate because, as explained in the introduction, the polyol groups form the soft segments. Thus, in simple terms, the differences in adhesion are due to the proportion and structure of the soft segments of the TPUs.

The influence of the proportion of the respective soft segments can be clearly observed in the results of Ether 85A compared to the two harder materials Ester 95A and Ether 95A. There is a percentage difference between the two hard materials and the material with a higher proportion of soft segments that is significantly greater than the difference between Ester 95A and Ether 95A. This observation is also supported by the high bond strength of Ester 75A, which has an even higher proportion of soft segments than Ether 85A.

There are many reasons why a higher proportion of soft segments promotes adhesion formation. As mentioned above, the soft segments in TPUs consist of long‐chain diols. Unlike the hard segments, these chains are very flexible. Especially when cyclic compounds are present in the hard segments, as is the case with aromatic diisocyanates, the chain mobility also decreases. As the polymer chains become more mobile with a higher proportion of soft segments, the diffusion process is accelerated and there is a higher proportion of entanglements. This increases the thickness of the interfacial layer and leads to an increase in bond strength. Another aspect of more flexible chains is the possibility of better convergence. The more flexible a material is, the better it can conform to a given shape. This also applies to the macromolecules, which can better conform to the macromolecules of the PLA‐based substrate material. Greater conformability promotes the formation of secondary valence forces, as these have small ranges, which in turn has a positive effect on composite adhesion and various adhesion theories.

In addition, the density of the second component also appears to affect bond strength. The density of the TPUs is 1.17, 1.21, 1.12, and 1.15 g/cm^3^ for Ester 75A, Ester 95A, Ether 85A, and Ether 95A, respectively (specifications of the manufacturer). It can be seen that the density of Ester 75A is lower than that of Ester 95A and that the density of Ether 85A is lower than that of Ether 95A. Therefore, the TPUs with the lower densities and higher soft segment content appear to form a better bond. The harder TPUs with a higher proportion of crystalline hard segments have a higher density, as this increases with the degree of crystallinity [92]. This confirms the statement that better adhesion is induced by a higher proportion of soft segments, as their degree of crystallinity is lower.

Not only the amount of soft segments in the TPU, but also their structure affects bond strength. With a few exceptions, almost all Ether 95A batches show higher strengths than those of Ester 95A. The proportion of soft segments in these two TPUs must be similar because they have the same Shore A hardness. Looking at the structures of polyester and polyether separately, polyester appears to be more branched than polyether, mainly due to the C=O bond. As a result, a soft segment of the polyether may be present in a more straight‐chain form, resulting in a better approximation of the second component to the substrate material. According to adsorption theory, a high degree of contact between the two materials is necessary for adhesion. By bringing the polymer chains of the first and second components closer together, the intermolecular forces can have a better effect due to their limited range. In addition, the number of entanglements can be increased. The result is a bond with better adhesion.

These two observations show that the higher peel strength of Ether 85A is due to the polyether polyols and the proportion of soft segments. However, when looking at the effects of the different types of storage, it is noticeable that Ether 85A has a significantly higher loss of adhesion in all types of storage than the other materials. This is best explained by the weak boundary layer theory, as the other theories used to describe the adhesion of 2C composites cannot explain the large loss compared to the reference. All types of storage have a similar effect on the peel resistance of Ether 85A. Therefore, it is reasonable to assume that low‐molecular‐weight impurities accumulate at the interface as a result of storage, causing or promoting the separation of the two components. This can occur, for example, through thermal or hydrolytic degradation products on the material surfaces. This assumption is confirmed by the fact that there are no major differences between the effects of each storage condition and the reference on the mechanical properties. The small changes in tensile strength and elongation at break were largely positive, demonstrating that there is no correlation between the change of mechanical properties and the change in adhesion force.

Although the other materials also show a loss of adhesion due to the different storage types, this loss of adhesion is not nearly as significant and can also be attributed to different adhesion theories. In principle, a deterioration in peel strength from the reference is to be expected for any type of storage, since each type of storage adds mass or energy to the specimen in some way, and this influences the boundary layer. A distinction must be made between the influence of temperature and the influence of relative humidity. Without any influence, the reference measurement values of the test specimens are nearly constant for all specimens over the investigated period of 2 weeks.

It is unlikely that the effect of temperature has caused sufficient damage to the material to explain the loss of peel strength, as the mechanical properties of the TPU do not deteriorate during the tensile tests performed, but in some cases even increased compared to the reference. The results of the DSC measurements show that temperature storage led to an increase in crystallinity, which explains the increase in tensile strength.

In addition to the effects on mechanical properties, the storage‐related effects also affect adhesive properties. For example, the aging phenomena can weaken diffusional entanglements of the polymer chains through chain scission or movement and neutralize or regroup adhesion‐promoting polar edge groups. The decrease in polar edge groups was confirmed by FTIR‐ATR measurements. In addition, the post‐crystallization mentioned above affects the interfacial tension between the two materials through a change in the SFE and possible shrinkage effects. The consequences of these effects are evident in the peel strength of all temperature‐stored materials relative to their reference. Another possible cause may be the increasing permeation coefficient of TPUs with increasing temperature [93]. This would promote the penetration of gases into the boundary layer and thus provide a link to the weak boundary layer theory. However, it should be noted that only the effects of storage on the TPUs and composites were investigated, not those on the PLA‐based substrate material. However, it is expected that all material combinations will show comparable changes as a result of storage.

For all changes attributable to the two temperature effects, it was found that the various effects were noticeable within 1 day and that longer storage had little effect. It was expected that an increase in temperature from 60°C to 80°C would increase the degradation of bond strength. This is true at least for the two materials Ether 85A and Ether 95A. The fact that a higher temperature has a greater effect on the material can be explained by the theories mentioned above, as their mode of action is increased and the effect is more pronounced. However, this is not the case with Ester 95A, as temperature has less of a negative effect on adhesion. This is supported by the fact that a temperature difference of 20°C has almost no effect. In some cases, the peel resistance at 80°C was even higher than at 60°C. This observation correlates with the tensile test results of Ester 95A. There, the tensile strength of both ether‐based TPUs remained nearly constant between 60°C and 80°C, whereas the tensile strength of Ester 95A increased again between these two temperatures. It is therefore reasonable to assume that the improvement in bond strength in the peel test of Ester 95A was initiated by the change in the properties of the TPU.

Looking at the effect of relative humidity on the specimens (with the exception of Ester 75A), it can be seen that Ester 95A has the lowest peel strength and Ether 85A has the highest, regardless of the length of storage time, with Ether 95A in between. Since polyether polyols generally have better hydrolysis resistance, the question arises as to whether this is also important for the composite. In principle, the ether‐based TPUs are better in absolute terms in this study. However, the loss of adhesion compared to the reference is similarly high for the two harder TPUs with a Shore A hardness of 95 and is around 40%. Therefore, the higher hydrolytic resistance of Ether 95A compared to Ester 95A cannot increase the resistance of the bond strength. In addition, the tensile tests of these two materials confirm this statement, as a similar loss of tensile strength is documented, and the material with the polyether polyols is affected by storage at elevated humidity in the same way as the ester‐based material. Considering the increase in weight of all TPUs tested and the effects just described, it can be assumed that no or only very little hydrolytic aging effects occurred due to storage and that the changes in mechanical properties and bond strength were caused solely by water absorption.

The TPUs also behave similarly in the mechanical tests after storage in a climatic chamber at 80°C and 80% relative humidity. On the one hand, the materials lose tensile strength; on the other hand, the elongation at break increases only during this storage in direct comparison with the reference (exception: Ester 75A). The higher temperature of 80°C compared to room temperature seems to make a difference during humidity storage. The observed increase in elongation at break can be related to the water absorption of the material, which is confirmed by the observed increase in weight. The permeation coefficient, which is influenced by temperature, favors the penetration of water vapor, and the increased chain mobility ensures that more water molecules can accumulate in the material. Only in the case of Ester 75A is a significant weight reduction observed, as the plasticizer diffusion favored by the temperature increase outweighs the water absorption. This correlates with the observed reduction in elongation at break.

The combined storage of temperature and humidity caused the material bond to detach in all material combinations over the course of the storage period. After 3 days of storage, the majority of the adhesive bond was destroyed by these environmental conditions. In addition to the loss of the adhesive bond, which was characterized by the incorporation of water into the interfacial layer, there was also a decomposition of the substrate plate from the PLA blend. As a result, individual pieces adhered to the soft TPU tab without cohesion during removal. Therefore, although it was possible to perform the tensile tests on the TPUs after this storage, the low residual bond strength could no longer be tested. Therefore, for future studies, it is useful to either choose a more resistant material for the substrate plate or to perform the storage at lower temperatures. In addition, other ester and ether‐based TPUs with different Shore hardness should be considered to validate the effects observed here.

## 5 Conclusions

The aim of this study was to demonstrate the resistance of bio‐based TPUs based on polyester and polyether in bio‐based hard‐soft composites produced by a two‐component injection molding process to various environmental influences. For this purpose, both the bond strength and the mechanical properties were characterized as a function of the type and duration of storage. The environmental conditions were simulated by storage at 60°C and 80°C and at 80% relative humidity in combination with 23°C and 80°C.

The following results were obtained from the test series:

- All TPUs show good to very good adhesion to PLA‐based substrates and are therefore well suited for 2C composites with technical requirements. In the case of Ester 75A, the bond strength was even higher than the tensile strength of the TPU, resulting almost exclusively in cohesive failure of the TPU tab. The bio‐based nature of TPU is therefore demonstrably not an obstacle to the use of technical multi‐component components manufactured using 2C injection molding.
- The higher the proportion of the soft segment in the TPUs used in this study, the higher the adhesion, since the higher mobility of the long diol chains favors diffusion processes and brings the two materials of the composite closer together, causing secondary valence forces to act.
- The structure of the soft segment has a decisive influence on the adhesion of the materials used in this study. Polyethers are less branched and therefore allow the two materials to come closer together, favoring the adhesion‐enhancing effects described by the adsorption theory of adhesion.
- For all material combinations, exposure to elevated temperatures resulted in a decrease in bond strength, with the effects increasing with temperature for the ether‐TPUs, but not for Ester 95A. The changes in bond strength correlate with the changes in mechanical properties.
- The improved hydrolysis resistance of the ether‐based TPU does not affect the degradation of bond strength caused by storage in high humidity. Both the ester and ether‐based TPUs with a Shore hardness of 95 show a decrease in bond strength of approximately 40% as a result of storage in humid conditions. The results of the characterization of the mechanical properties as well as the increase in weight of the test specimens confirm that no hydrolytic aging effects occur during storage and that the observed effects are due to the absorption of water molecules.
- Combined storage at elevated humidity and temperature has the greatest effect on bond strength. A complete loss of bond strength occurred after only a few days, and no further tests could be performed.

## Conflicts of Interest

The authors declare no conflicts of interest.

## Data Availability

The data that support the findings of this study are available from the corresponding author upon reasonable request.
